# Recovering arm function in chronic stroke patients using combined anodal HD-tDCS and virtual reality therapy (ReArm): a study protocol for a randomized controlled trial

**DOI:** 10.1186/s13063-021-05689-5

**Published:** 2021-10-26

**Authors:** Camille O. Muller, Makii Muthalib, Denis Mottet, Stéphane Perrey, Gérard Dray, Marion Delorme, Claire Duflos, Jérôme Froger, Binbin Xu, Germain Faity, Simon Pla, Pierre Jean, Isabelle Laffont, Karima K. A. Bakhti

**Affiliations:** 1 Physical and Rehabilitation Medicine, Centre Hospitalier Universitaire (CHU) - Montpellier, Lapeyronie, 371 Avenue du Doyen Gaston Giraud, 34295 Montpellier, Cédex 15 France; 2 EuroMov Digital Health in Motion, Université Montpellier, IMT Mines Alès, Montpellier, France; 3 Silverline Research, Brisbane, Australia; 4 Physical and Rehabilitation Medicine, CHU Nîmes, Le Grau du Roi, France; 5 Clinical Research and Epidemiology unit, CHU Montpellier, Université Montpellier, Montpellier, France; 6 Health Directorate, CHU Montpellier, Montpellier, France; 7 Clinical Investigation Centre, CHU Montpellier, Montpellier, France - Inserm, CIC 1411, Montpellier, France

**Keywords:** HD-tDCS, Upper limb function, Virtual reality therapy, Stroke, Rehabilitation, Laterality index, Monitoring, Upper limb use, Cortical activation

## Abstract

**Background:**

After a stroke, 80% of the chronic patients have difficulties to use their paretic upper limb (UL) in activities of daily life (ADL) even after rehabilitation. Virtual reality therapy (VRT) and anodal transcranial direct current stimulation (tDCS) are two innovative methods that have shown independently to positively impact functional recovery of the paretic UL when combined with conventional therapy. The objective of the project will be to evaluate the impact of adding anodal high-definition (HD)-tDCS during an intensive 3-week UL VRT and conventional therapy program on paretic UL function in chronic stroke.

**Methods:**

The ReArm project is a quadruple-blinded, randomized, sham-controlled, bi-centre, two-arm parallel, and interventional study design. Fifty-eight chronic (> 3 months) stroke patients will be recruited from the Montpellier and Nimes University Hospitals. Patients will follow a standard 3-week in-patient rehabilitation program, which includes 13 days of VRT (Armeo Spring, 1 × 30 min session/day) and conventional therapy (3 × 30 min sessions/day). Twenty-nine patients will receive real stimulation (4x1 anodal HD-tDCS montage, 2 mA, 20 min) to the ipsilesional primary motor cortex during the VRT session and the other 29 patients will receive active sham stimulation (2 mA, 30 s). All outcome measures will be assessed at baseline, at the end of rehabilitation and again 3 months later. The primary outcome measure will be the wolf motor function test. Secondary outcomes will include measures of UL function (Box and Block Test), impairment (Fugl Meyer Upper Extremity), compensation (Proximal Arm Non-Use), ADL (Actimetry, Barthel Index). Other/exploratory outcomes will include pain, fatigue, effort and performance, kinematics, and motor cortical region activation during functional motor tasks.

**Discussion:**

This will be the first trial to determine the impact of adding HD-tDCS during UL VRT and conventional therapy in chronic stroke patients. We hypothesize that improvements in UL function will be greater and longer-lasting with real stimulation than in those receiving sham.

**Trial registration:**

The ReArm project was approved by The French Research Ethics Committee, (Comité de Protection des Personnes-CPP SUD-EST II, N°ID-RCB: 2019-A00506-51, http://www.cppsudest2.fr/). The ReArm project was registered on ClinicalTrials.gov (NCT04291573, 2^nd^ March 2020.

**Supplementary Information:**

The online version contains supplementary material available at 10.1186/s13063-021-05689-5.

## Background

With a very high prevalence, stroke is the second cause of death and the first cause of acquired disability in adults in well-developed countries. Stroke frequently leads to sensory-motor and functional physical limitations [32]. It is also responsible of 2 to 4% of total expenses in health care with a need of developing new re-educative treatments [32]. Hemiparesis is one of the most common disabilities caused by stroke. Poor recovery of the paretic UL function affects more than 80% of stroke survivors [26] and is mostly persistent 3 months post-stroke, in the late sub-acute and chronic stages, even after intensive rehabilitation [54]. Although, repetitive motor training appears to be efficient for improving paretic UL function [73], there is no standardized protocol for the rehabilitation of the paretic UL after stroke [60]. The lack of recovery often results in non-use of the paretic UL [68], leading to reorganization of brain functional networks [48] and finally restricting use dependent functional recovery [50].

Stroke leads to spontaneous reorganization of the central nervous system corresponding to neural plasticity [34]. Spontaneous recovery and regeneration mechanisms of brain reorganization are triggered soon after a brain lesion. Over time, from late sub-acute stage to chronic stage of stroke, there is less and less spontaneous plasticity and it is more difficult to recover paretic UL function [34]. During the late sub-acute and chronic stages after a stroke, experience-dependent plasticity takes on a greater role for functional recovery of the paretic UL [37]. Some aspects of the rehabilitation program (e.g., intensity and volume of practice, variety, specificity, motivation, and biofeedback) can modulate this experience-dependent plasticity [38, 65]. In this context, virtual reality therapy (VRT) that combines rehabilitation exercises with gaming elements appears to be a well-suited intervention in promoting functional recovery of the paretic UL [45].

VRT can be defined as the use of a brain-computer interaction method [70] for providing patients multimodal sensory and motor stimuli with the help of immersive environment workspaces. Herein, VRT allows a simulated practice of functional motor tasks with more repetitions than in conventional therapy used in post-stroke rehabilitation [19, 40, 70]. Furthermore, VRT can limit fatigue and loss of enthusiasm and improve patient cooperation and engagement, which can positively influence recovery of the paretic UL [42, 43, 67]. As such, biofeedback provided to the patient during VRT was shown to improve the activation of the lesioned hemisphere, fostering a better relearning of motor tasks [33]. A recent Cochrane review [45] indicated that VRT is equally effective in improving paretic UL function compared to conventional therapy (low-quality evidence); however, there was some evidence that VRT resulted in a slightly better ability to manage ADL such as showering and dressing (moderate-quality evidence). Moreover, when combined with a conventional therapy rehabilitation program post-stroke, VRT more efficiently improves paretic UL function and patient’s capability to succeed in ADL [41, 45]. Among the VRT methods available, robot-assisted VRT systems use an exoskeleton providing assistive control of the paretic UL (e.g., through counter-gravity springs, such as Armeo system) and thus allows longer training sessions with reduced fatigue [49]. Accordingly, robot-assisted VRT has been shown to achieve greater and long-lasting improvements in paretic UL function in comparison to conventional therapy alone [69].

Enhancing recovery of paretic UL function after stroke can also result from non-invasive brain stimulation (NIBS) through modulation of motor cortical excitability. NIBS has the ability to modulate neural excitability and generate new neuronal associations inducing in turn brain plasticity [62]. Among NIBS techniques, transcranial direct current stimulation (tDCS), which applies low currents around 1–2 mA during 10 to 30 min for single or multiple sessions [10, 15] is a promising clinical potential that can be easily added onto an UL rehabilitation protocol. Moreover, its active sham mode enabling randomized clinical trials allows to apply brief stimulation (10–90 s) while the patient is receiving a rehabilitation treatment that provides the same scalp sensations as a real stimulation session [61, 63]. By increasing ipsilesional motor cortical excitability and promoting neuroplasticity, anodal tDCS added to motor task training has been shown to improve functional recovery of the paretic UL after a stroke [1, 23–25, 70]. Allman et al. [1] reported long-term (3 months) improvements in functional paretic UL clinical assessments (Wolf Motor Function Test (WMFT), Action Research Arm Test (ARAT)) in chronic stroke patients (> 6 months) with ipsilesional anodal tDCS added to daily hand and arm motor rehabilitation training over 2 weeks (9 × 1-h sessions) compared to sham stimulation. This study also showed an increased movement related cortical activation and grey matter volume in ipsilesional motor cortical regions for patients in the stimulation group compared to sham. Figlewski et al. [23] applied anodal tDCS to the ipsilesional motor cortex during constraint-induced therapy in chronic stage stroke patients (3–36 months) over 2 weeks (9 × 6-h sessions) and found greater improvements in paretic UL function (WMFT) when compared with constraint-induced therapy alone. A recent Cochrane review [22] indicated the effectiveness of tDCS versus sham for improving ADL outcomes after stroke (low to moderate quality evidence). Altogether, these studies provide clinical support that adding anodal tDCS to a rehabilitation program may bolster more the recovery of paretic UL function and ADL in chronic stroke patients. Indeed, anodal tDCS might facilitate activity-dependent plasticity within the neural networks recruited already within a rehabilitation treatment, thereby contributing to greater and sustainable clinical gains [1, 11, 23].

The two aforementioned adjunct stroke rehabilitation methods (VRT and anodal tDCS), used separately as a complement to conventional rehabilitation are expected to improve and maintain recovery of the paretic UL by positively impacting neuroplasticity. Thus, to enhance the effect of conventional therapy, adding anodal tDCS during VRT could be more efficient than when VRT is used alone [66, 70]. However, there is a paucity of studies that have evaluated the effectiveness of this VRT and anodal tDCS combination in post-stroke rehabilitation. Triccas et al. [71] investigated the effects of adding anodal tDCS (ipsilesional motor cortex, 1 mA, 20 min) to an 8-week robot-assisted VRT (Armeo spring exoskeleton) rehabilitation program (18 × 1-h sessions) for early sub-acute (2–3 months) and chronic (9–61 months) stroke patients. Although the addition of anodal tDCS to VRT was well tolerated, there were no added clinical benefits (Fugl-Meyer Assessment, ARAT, Motor Activity Log (MAL), Stroke Impact Scale (SIS)) of such a combination. The authors considered that the low number of sessions of VRT per week (2–3 sessions) and the small/heterogenous group of patients per group (*n* = 11) might have reduced the chances of detecting a clinical difference between groups. Moreover, the standard anodal tDCS montage used could prevent significant changes of excitability within the target brain regions. Improving the focality of tDCS effects over sensorimotor regions with anodal high definition (HD)-tDCS was found to induce a greater increase in cortical excitability [39] and haemodynamic activity [55] comparatively to conventional tDCS montage in healthy subjects and thus, might induce a greater neuroplasticity stimulus. Chhatbar et al. [17] reported from a meta-analysis of tDCS UL motor recovery studies a dose–response relationship with electrode size and current density, such that a higher current density or smaller electrode size being associated with greater efficacy on paretic UL motor recovery (Fugl Meyer Upper Extremity (FM-UE)) in chronic stroke. Therefore, it can be expected that applying a more intensive anodal HD-tDCS (2 mA, 20 min) and VRT program (3 weeks, 13 × 30-min sessions) will provide greater clinical benefit [21].

The ReArm project is a randomized-control trial of late subacute and chronic stroke patients (> 3 months) designed to determine the impact of adding anodal HD-tDCS (2 mA, 20 min, ipsilesional motor cortex), compared to sham, during an intensive 3-week UL VRT program (13 × 30-min sessions) supplementing a conventional therapy program (39 × 30-min sessions). The primary objective will be to evaluate the impact of this intervention the on paretic UL function 3 months after the end of the rehabilitation. Secondary and exploratory objectives will be to investigate the evolution of the paretic UL use in ADL, performance, kinematics, and movement-related activation of motor cortical regions during functional motor tasks, from the beginning to the end of the 3-week program and after 3 months. We hypothesize that adding anodal HD-tDCS during VRT, as a supplement to conventional therapy rehabilitation program, will result in greater retention of clinical benefits 3 months after the intervention and increased use and functional recovery of the paretic UL.

## Methods

### Ethics

The study was approved by the National Research Ethical Committee (Comité de Protection des Personnes-CPP SUD-EST II, No. ID-RCB 2019-A00506-51, http://www.cppsudest2.fr/) and is registered on http://www.clinicaltrials.gov (NCT number, NCT04291573).

### Participants

Fifty-eight adult hemiparetic stroke patients will be recruited (see “Sample size” section for details).

#### Inclusion criteria

Inclusion criteria will be patients (1) aged between 18 and 90 years old, (2) at more than 3 months of a first cerebrovascular accident of any aetiology (haemorrhagic or ischaemic), and (3) with UL motor impairment (FM-UE ≥ 15 [27]).

#### Exclusion criteria

Exclusion criteria will be patients (1) that fail to give written informed consent, (2) with uncontrolled epilepsy, (3) with pacemaker or any metallic object implanted in the brain, (4) who are pregnant or breastfeeding, (5) that have hemineglect or severe attentional problems (omission of more than 15 bells on the Bell’s test [29]), (6) with aphasia of comprehension dysfunction (Boston Diagnostic Aphasia Examination < 4/5 [36]), (7) with severe cognitive dysfunction (Mini Mental State Examination-MMSE < 24 [35]), (8) not covered by a French social security scheme, and (9) under guardianship.

#### Settings and recruitment

Patients will be recruited from in-patient and out-patient clinics at the physical and rehabilitation medicine (PRM) departments of the two university hospitals (CHU): Lapeyronie (Montpellier, France) and Nîmes (Grau du Roi, France). Before including patients in the ReArm protocol, the medical physician taking care of the patient will check if the patient fits with the inclusion criteria and does not present any exclusion criteria during a usual clinical outpatient visit. The adherence of patients will be improved by an in-patient rehabilitation stay (patients will sleep at the hospital during training days). The investigators will be available any time to explain to the patient the objective of this project.

### Study design

**Fig. 1:**
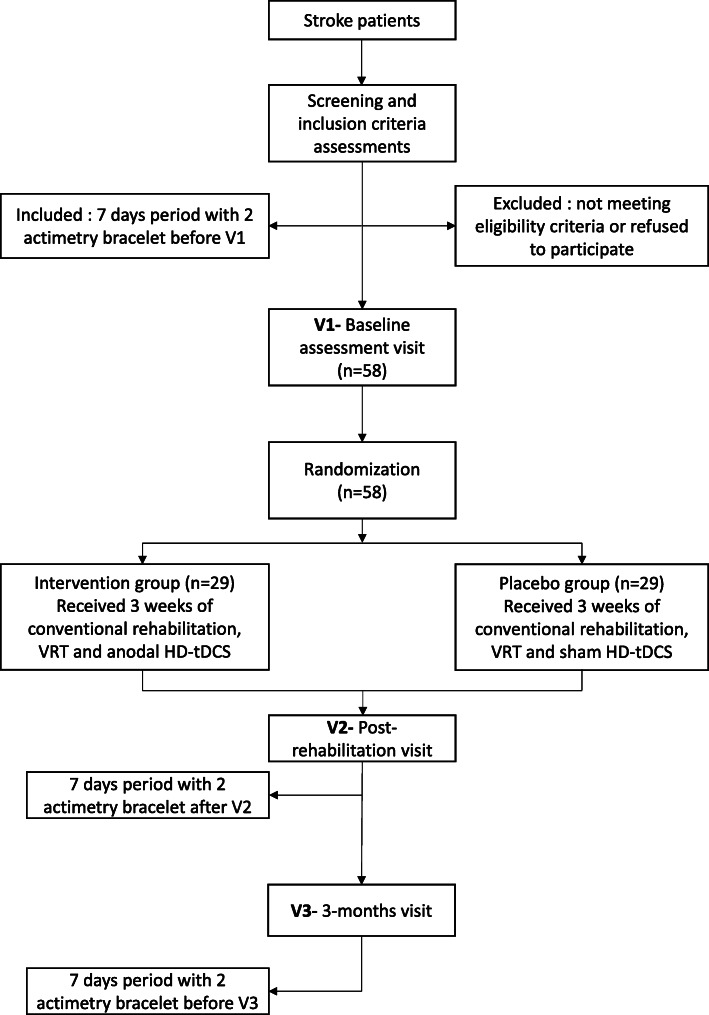
Flow chart of the study design

Figure 1 shows a flow diagram of the study design. The ReArm project is a quadruple-blinded, randomized, sham-controlled, bi-centre, two-arm parallel, and interventional study. All patients will undergo 3 weeks of a standard in-patient stroke rehabilitation program (see details in the next section) and will thereafter be followed over a period of 3 months. One group (intervention) will receive 3 weeks rehabilitation consisting of standardized conventional therapy rehabilitation and combined VRT and anodal HD-tDCS. The second group (placebo) will receive standardized conventional therapy rehabilitation and combined VRT and sham HD-tDCS.

#### Randomization

The Capture System software (Ennov Clinical, module randomization, Paris, France) will be set up by the randomization administrator, from the Innovation and Research Department of the Hospital, to give a blinding code to each patient. A centralized randomization will be performed using the Capture System software (Ennov Clinical, module randomization, Paris, France). A minimization algorithm will assure the balance of groups regarding PRM centres (CHU Montpellier or Nimes) and stroke severity (FM-UE score lower or higher than 40 [72]); see “Blinding of group and stimulation protocols” section for more details. For each inclusion, another hospital department, the clinical investigation centre, will use the software to allocate the right protocol.

### Standard in-patient stroke rehabilitation program

Both the intervention and placebo groups will follow the standard in-patient stroke rehabilitation program usually proposed to stroke hemiparetic patients at the Montpellier and Nimes PRM Departments, which includes both conventional therapy and VRT sessions on each day of a 3-week in-patient stroke rehabilitation program. In both PRM departments, VRT has been, since 2014, included in the standard in-patient stroke rehabilitation program.

#### Conventional therapy sessions

Conventional therapy includes physiotherapy (1 × 30 min) and occupational therapy (2 × 30 min) sessions spaced over the morning and early afternoon each day. Each session is adapted to the fatigability and recovery of each patient. The physiotherapy session focuses mainly on trunk balance, sensorimotor exercises, standing balance and walking workout, and muscular and cardio-respiratory reconditioning to effort. The occupational therapy sessions are oriented towards sitting and standing balance workout, sensorimotor exercises for movement restoration, and may include prehension exercise, and mirror therapy. A daily speech therapy session will be added when required (language troubles).

#### Virtual reality therapy (VRT) sessions

In addition to the conventional therapy sessions, each day, patients will receive a 30-min VRT session using an UL exoskeleton (ARMEO®, HOCOMA, Volketswil, Switzerland) providing spring-based anti-gravity support. The ergonomic and adjustable exoskeleton embraces the whole UL, from shoulder to hand and counterbalances the weight of the patient’s UL. The Armeo spring VRT system enables hemiparetic patients to achieve more movements in a 3-dimensionnal workspace than they could usually do without anti-gravity support [53, 69]. The Armeo exoskeleton is connected to a computer displaying meaningful functional tasks (e.g., cleaning a window) on a computer screen. Sensors record the active movements and all joint angles during the therapy session. This semi-immersive VRT provides auditory and visual feedback during and after games training. The virtual environment allows to change the type of games and their difficulty but the main objective of all the games is focused on mobilization of the entire paretic UL with some games also including pro/supination (e.g., flower watering) and grasping with the paretic hand (e.g., egg cracking).

**Fig. 2:**
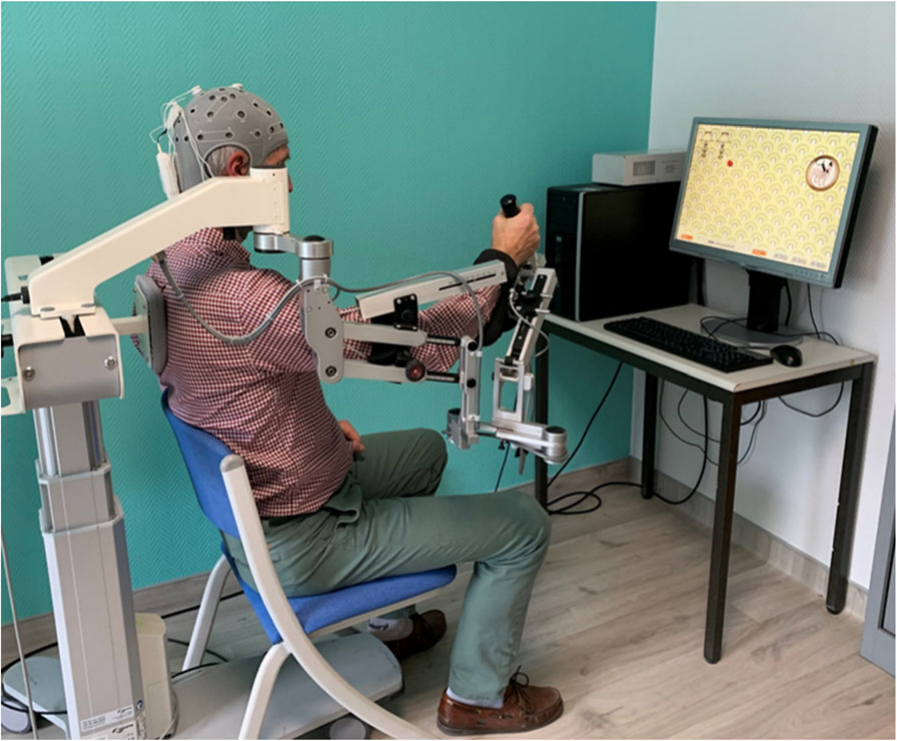
Patient setup on the ARMEO virtual reality therapy system. The ARMEO exoskeleton counterbalances the weight of the patient’s upper limb with springs located on the elbow and wrist joints. The patient in a resting position, ready to begin to play the ladybug game

Initially, the patient is comfortably seated on the Armeo system; the display screen is placed 75 cm from the patient and the centre of screen 20 cm below the eyes (Fig. 2). The parameters of the ARMEO® (e.g., exoskeleton arm and forearm lengths, arm and forearm anti-gravity compensation, auto-grip), will be set for each patient on the first day (V1) and will not be changed for subsequent sessions. To counterbalance the weight of the patient’s UL, the upper arm and forearm weight compensation is half for all patients. The UL is straight forward if the patient does not move (rest position).

**Fig. 3:**
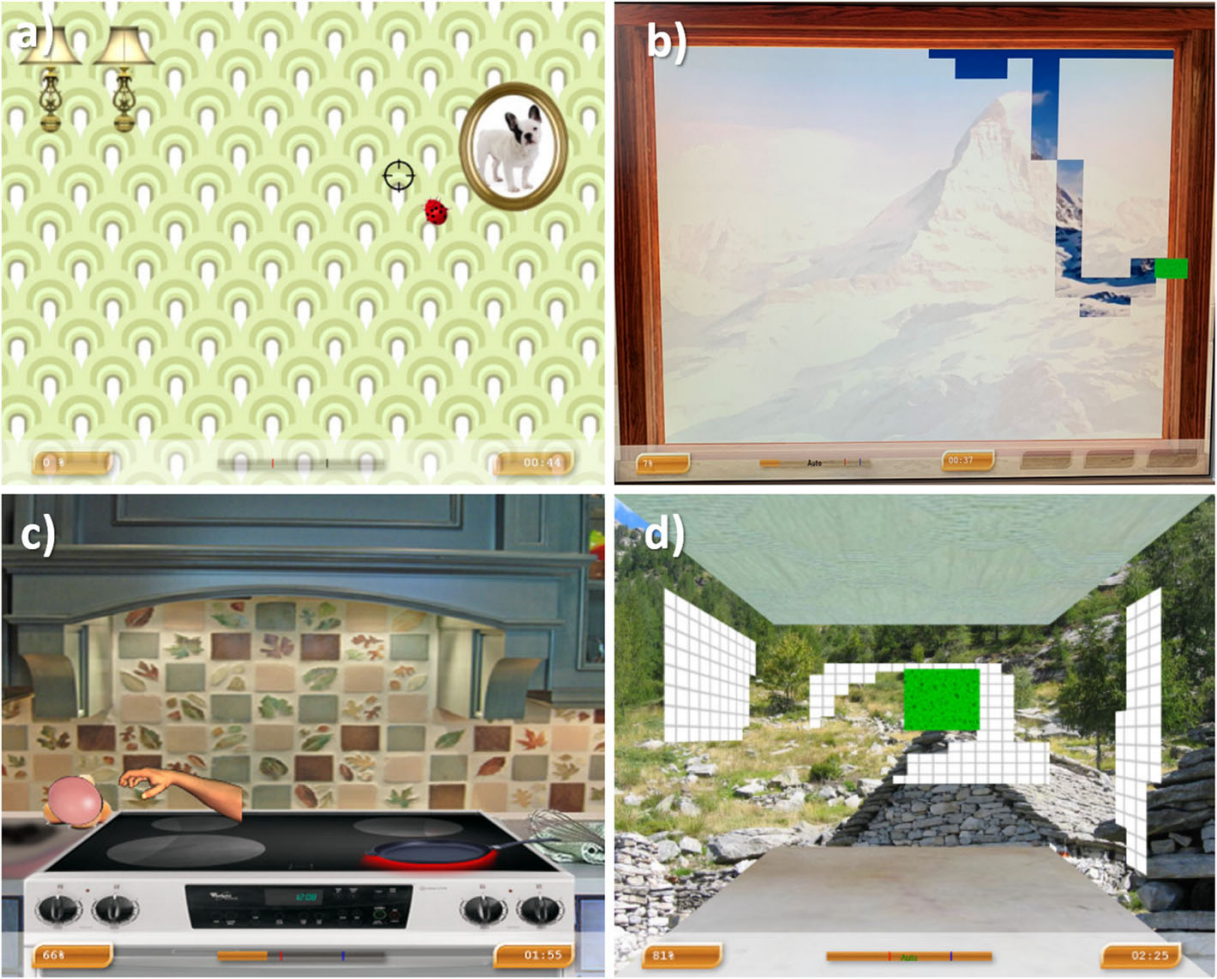
Examples of ARMEO virtual reality therapy games. **a** Ladybug requires the patient to “catch” a series of 48 Ladybugs appearing one after the other on different parts of the screen with a time limit of 3 min. **b** Windows Mopping requires the patient to clean a window by moving the virtual sponge in 2D workspace to completely reveal a panorama. **c** Egg cracking requires the patient to carefully grasp an egg from a virtual pot and move it to a stove. **d** Reveal panorama requires the patient to move a virtual sponge in 3D workspace to completely reveal a panorama

Figure 3 shows 4 examples of the Armeo VRT rehabilitation games. For all patients, each session will start and end with the *Ladybug* game (Fig. 3a), a speed-accuracy test where the patient has to point to the ladybugs (targets) appearing on the screen as fast as possible. The rehabilitation games during the first 20 min of the session will remain identical for all subjects, with the same program order of 9 rehabilitation games (see Table 1). These 9 games enable patients to use any remaining UL motor function (shoulder, elbow, hand) in a 3D workspace. The 9 games include one 3D game (Reveal Panorama), seven 2D games in frontal or horizontal plane (window mopping, egg cracking, fish catching, popping air bubbles, fruit shopping, stove cleaning, reaction time) and one 1D game (snow balls). The next 10 min will include rehabilitation games selected specifically to each patient’s preferences and abilities (e.g., pro/supination). The therapeutic plan for the final 10 min of the 30-min session (order and type of exercises) will be decided during the first session by the therapist in charge of the patient (trying games with them). Patients will be asked to provide feedback after the first session concerning the difficulty of the games, their ability to achieve them, and their preferences. This will allow the therapist to choose the games of the last 10 min of the session. Each patient will follow the set rehabilitation games program configured to their ability that will remain identical for all subsequent sessions.

**Table 1:** List of the ARMEO virtual reality therapy games detailing the upper limb joints, movements, and workspace

Name of the games	Joints				Workspace/plane
	Elbow ext./flex.^a^	Shoulder ext./flex.	Shoulder horiz abd./add.^b^	Hand (grasp)	
Windows mopping	X	X	X	X	2D/frontal
Reveal panorama	X	X	X	X	3D/frontal + horizontal
Egg cracking	X	X	X	X	2D/frontal
Fish catching	X	X	X		2D/frontal
Popping air bubbles	X	X	X	X	2D/frontal
Snow balls	X	X			1D/horizontal
Fruit shopping	X	X	X	X	2D/frontal
Stove cleaning	X	X		X	2D/horizontal
Reaction time	X	X	X		2D/frontal

^a^Extension/flexion

^b^Horizontal abduction/adduction

The difficulty of the rehabilitation games will be initially set at the highest level (e.g., smallest size of sponge for the window mopping game, see Fig. 3b) in order to allow improvements in performance over the training sessions. Moreover, if a patient achieves a maximum score of 100% on a game with a time limit, they will be able to further improve their performance reducing the time of the game which will be recorded. Depending on the paretic hand recovery level of the patient, auto-grip will be activated or not. Auto-grip is not activated for patients able to grasp. However auto-grip is activated when the patient just needs to move the UL to the location of the target without requiring a hand grip force to secure the target. The auto-grip will be modified to be off during the rehabilitation program if the hand grip ability of the patient improves. The Armeo software presents online and subsequently records each participant’s performance for each rehabilitation game (i.e., time, game performance). The software also records the kinematic movements of the exoskeleton, which will be used for the ladybug end effector trajectory analysis.

#### Anodal high definition transcranial direct current stimulation (HD-tDCS)

**Fig. 4:**
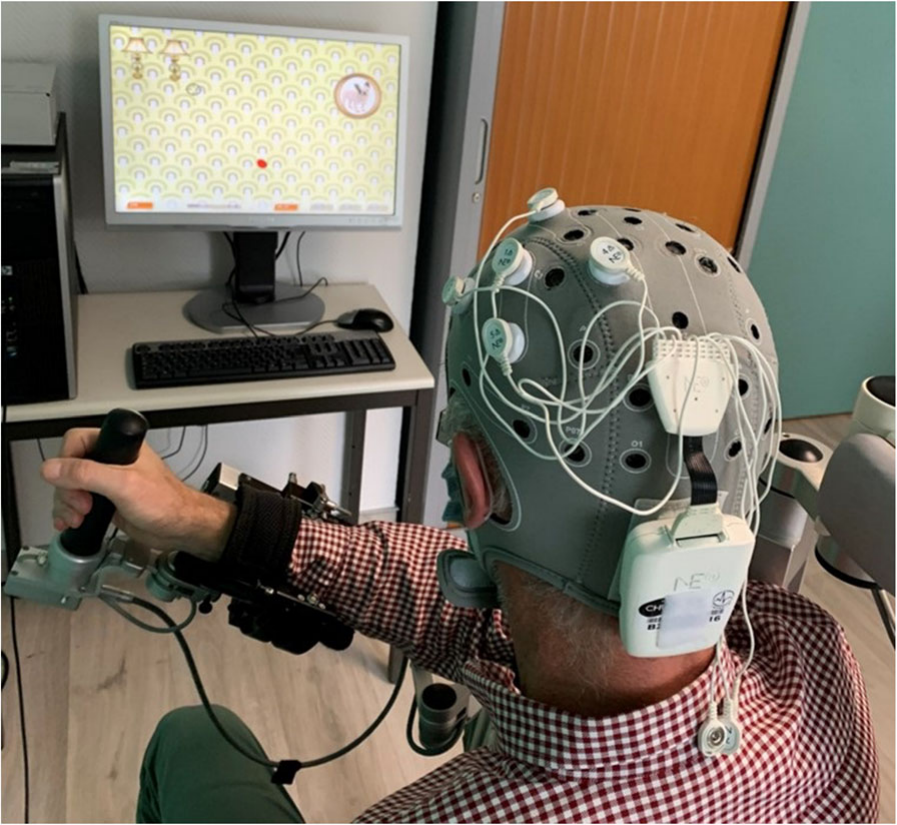
Anodal HD-tDCS applied to the ipsilesional primary motor cortex during an ARMEO virtual reality therapy session using the right upper limb. The patient is setup on the ARMEO exoskeleton equipped with the HD-tDCS cap comprising a Starstim NIC-power box delivering the current on the back and 5 electrodes located on the left primary motor cortex in a 4x1 HD-tDCS montage with anode on the C3 location

Real tDCS will be delivered to patients randomized in the intervention group (*n* = 29) using the Starstim8 device (Neuroelectrics, Barcelona, Spain). The Starstim8 will deliver a constant direct current to the motor cortex of the ipsilesioned hemisphere using a 4x1 HD-tDCS montage [8, 9, 13, 55]. The active anode electrode will be located on the scalp of the ipsilesional motor cortex (C3 or C4 of the 10–20 system) surrounded by four return electrodes (cathode) located at ~ 4 cm from the anode electrode (Fig. 4). The five HD-tDCS electrodes (NgPiStim, AgCl, 3.14 cm^2^) will be held in place on the scalp using a neoprene cap and each electrode will be filled with electrolyte gel (Signagel, Parker Laboratories, New-Jersey, USA). Patients will receive anodal HD-tDCS (2 mA) during the first 20 min of the VRT session. Current will ramp up from 0 to 2 mA for 30 s, remain constant for 20 min, and ramp-down to 0 for 30 s.

Patients randomized in the placebo group (*n* = 29) will wear the same equipment during the VRT session and will receive active sham HD-tDCS where the current will ramp up from 0 to 2 mA during the first 30 s, remain constant at 2 mA for 30 s, then ramp-down to 0 during the next 30 s, and remain at 0 for the next 20 min [9, 13]. This kind of active sham procedure provides the same feelings on the scalp as the real HD-tDCS session [8, 9, 55], which has been shown to be an optimal method for blinding of the stimulation [28, 56].

Before and after each 30-min VRT session (real or sham), subjects will be asked a few questions relating to the sensations on the scalp, which will be part of the pain and fatigue assessments (see “Secondary outcome measures” section). More specifically, patients will be asked if they feel any headache, stinging, itch, burn, and pain sensation on the scalp region where the HD-tDCS electrodes were located [8, 9].

#### Blinding of group and stimulation protocols

The randomization administrator of the project will designate 8 stimulation protocols in the NIC2 computer software controlling the Starstim8 device (4 active and 4 sham) and give them a blinded code (A to H). Only the blinded code of the program will be given to the therapist in charge of the rehabilitation to be launched for a specific patient. The Starstim8 device will be set in double-blind mode using the NIC2 software, so that the protocol panel does not show the type of stimulation (active or sham). This way, neither the patient, therapist, investigator, nor outcomes assessor will know which protocol is delivered (quadruple blind study). In order to assess blinding, patients will be asked at the end of the 3 weeks of rehabilitation if they think they were in the real or sham stimulation group. If needed (e.g., severe adverse events), blinding could be unblinded if the patient or the medical staff send a request to the Innovation and Research Department (see “Data management” section for more details).

### Description of testing visits and outcome measures

Table 2 presents the timing of the testing visits and outcome assessment measures. The baseline assessment (pre-test) visit (V1) will take place on the first day of the 3-week in-patient hospital rehabilitation stay. A specialist trained therapist will run the clinical assessments (WMFT, FM-UE, Box and Block Test (BBT), Bell’s test, MMSE, Barthel Index (BI); more details in the “Clinical data and outcome measures” section). Patients UL kinematics and motor cortical region activation will then be evaluated by one of the investigators on three functional motor tasks: a seated reaching task (Proximal Arm Non-Use, PANU), a circular steering task, and the Ladybug game of the Armeo VRT platform used for rehabilitation. The next day (day 2), patients will start the standard in-patient stroke rehabilitation program that will last for 13 complete days (excluding weekends) over 3 weeks.

**Table 2:** Study testing visits (V1-3) and outcome assessments

Outcome measures	Inclusion visit	Pre-test (V1)	Post-test (V2)	Retention test (V3, 3 months after V2)
Mini Mental State Examination	X			
Boston diagnostic aphasia examination	X			
Bell’s test	X			
Wolf Motor Function Test		X	X	X
Fugl-Meyer Upper Extremity		X	X	X
Box and Block Test		X	X	X
Barthel Index		X	X	X
Actimetry		X	X	X
Proximal Arm Non-Use (PANU)^a^		X	X	X
Reaching task (Kinematics and EEG/fNIRS)		X	X	X
Circular steering task (Kinematics and EEG/fNIRS)		X	X	X
Lady bug test (Kinematics and EEG/fNIRS)		X	X	X

^a^Proximal Arm Non-Use will be assessed during the reaching task

*EEG* Electroencephalography, *fNIRS* Functional near-infrared spectroscopy

The post-test visit (V2) will take place the last day of the 3 weeks (day 19); the post-test will be identical to the pre-test. The 3-month visit (V3) will take place 3 months after the end of the rehabilitation program (day 19) for a retention test and will be identical to the pre- (V1) and post- (V2) tests.

Patients will be given actimetry bracelets to be worn on each UL to measure UL activity for 7 days before V1, for 7 days after V2, and for 7 days after V3.

### Clinical data and outcome measures

#### Clinical data

Clinical data will be collected by the medical doctor during the routine clinical visit, including date of birth, age, sex, hand laterality before and after the stroke, date of the stroke, delay from the stroke to the inclusion, and characteristic of the brain lesion (type and location). Treatment, medication, and health events will also be reported. The source documents are the original data, sheets, and files, from which the data concerning the research are copied in the electronic case report form (eCRF) by the clinical investigation centre (CIC).

#### Primary outcome measure

##### Wolf Motor Function Test (WMFT)

The Wolf Motor Function Test (WMFT [74]) quantifies the motor ability of the UL through 15 timed and function based tasks (i.e., holding a pen, collect paper clips, prehension test) and 2 strength based tasks (arm lifting and hand grip). The speed at which functional tasks can be completed is measured by performance time and the movement quality when completing the tasks is measured by functional ability. The maximum time allowed to complete an item is 120 s. For functional ability scoring, a 6-point ordinal scale is used where 0 corresponds to “does not attempt with the involved arm” and 5 corresponds to “arm does participate/movement appears to be normal”. The WMFT thus gives four scores: (1) a sum of the function score reflecting the movement quality (0–75), (2) a median time score measuring the movement speed (0–120 s), and (3) arm lifting strength (Kg) and (4) hand grip strength (Kg). For our primary outcome analysis, the mean WMFT time and functional ability change scores between the two groups need to be 1.5–2 s for the timed score and 0.2–0.4 points for the functional ability score to be regarded as a clinically important difference [47].

#### Secondary outcome measures

##### Fugl-Meyer Upper Extremity test (FM-UE)

The motor deficit of the UL will be assessed using the FM-UE [27]. The FM-UE score allows to differentiate proximal UL movements (shoulder, elbow, and forearm; score 44) and distal UL movements (wrist and hand; score 24) totalling 66 points. The results of the scoring system of the FM-UE enable the classification of the patient as having mild (≥ 40/66), moderate (between 21/66 and 39/66) or severe (≤ 20/66) UL impairment. The FM-UE has excellent reliability, validity, and sensitivity to change and it is regarded as the gold standard to assess UL impairment [30]. The estimated clinically important difference of the FM-UE scores ranges from 4.25 to 7.25 points [57].

##### Box and Block Test (BBT)

The global hand grasping capacity will be assessed using the BBT [52]. The BBT requires the patient to move as many 2.5 cm^3^ cubes in 60 s (with only one cube been displaced at the time) from a box compartment to another separated by a 15.2-cm-tall partition using the paretic hand and then with the non-paretic hand. Only one trial is given. The patient score is equal to the number of these cubes transported in 60 s. For healthy subjects, the score at the BBT is between 75 and 80 cubes displaced in a minute. For our population, the smallest real difference before and after intervention is of five or six cubes [14].

##### Barthel Index (BI)

The activities of daily living will be assessed with the Barthel Index (BI [51]). BI assesses the patient’s level of independence to perform ten basic life activities: eating, bathing, personal care, ability to dress themselves, motility, urinary regularity, bathroom use, chair-bed transfer and inversely, mobility, and climbing stairs. The score generated varies from zero (totally dependent) to a maximum score of 100 (totally independent).

##### Actimetry assessment of paretic UL use in ADL

The actual use of the paretic UL in ADL in their own house environment will be assessed continuously over a 7-day period (prior to V1; after V2; after V3) with two wrist worn actimetry bracelets (AX3, Newcastle Helix, UK) on the paretic and non-paretic UL [3]. The two actimetry bracelets record 3D acceleration (± 8 G, 50 Hz) to get objective measures of the actual functional use of the paretic UL in the activities of daily living. The different measures will be the UseRatio (functional use ratio between the two UL), the Use (number of functional movements by the paretic UL), the intensity of paretic UL movements, and the actimetry profile of the different activities using the paretic UL [3, 44, 46, 58].

##### Proximal Arm Non-Use (PANU) score

The non-use of the paretic UL will be quantitatively assessed by the Proximal Arm Non-Use (PANU) score (0–100% [4, 5]) during a seated arm reaching task using kinematic measurements (see “Functional motor tasks” section for details). The PANU score quantifies the shoulder-elbow joint non-use that a patient can voluntarily cancel [5] and has good reliability and repeatability [4]. The PANU score of 6.5% is the limit beyond which we can consider the patient having proximal arm non-use [5].

**Fig. 5:**
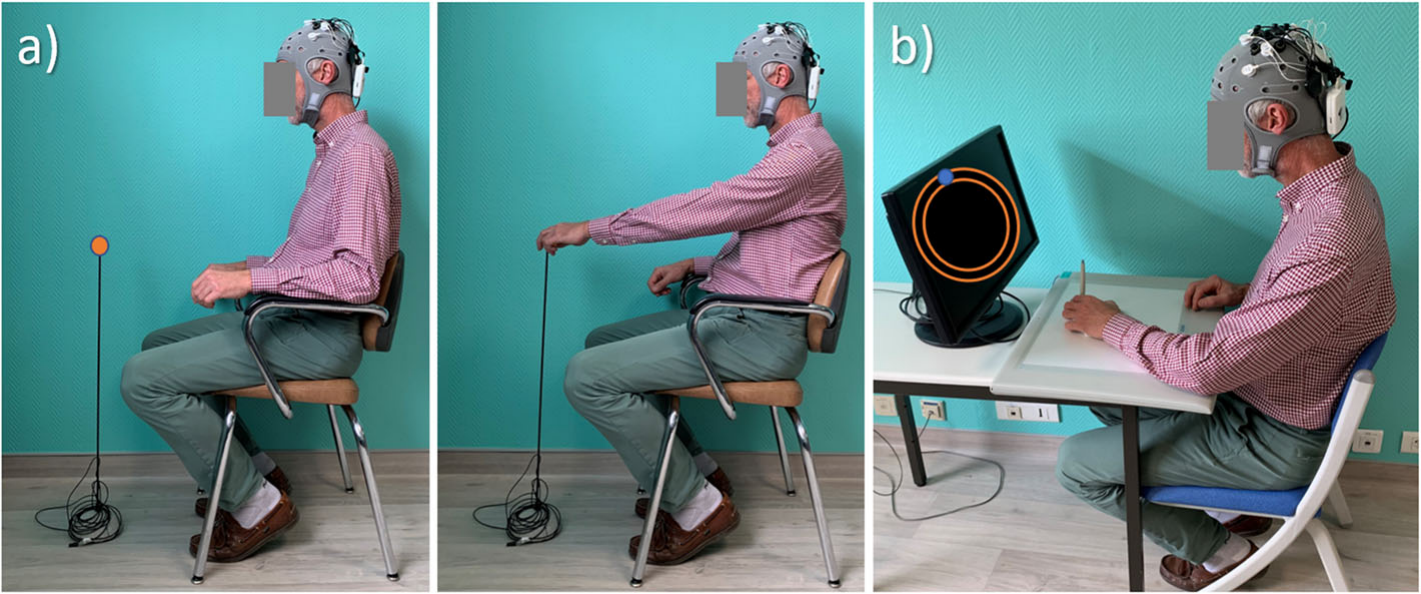
Functional motor tasks. Seated reaching task with the patient at rest and UL extended to reach towards the target (**a**). Circular steering task with the patient viewing the circle tunnel on the screen and moving the mouse cursor with the hand within the boundaries of the circle tunnel (**b**). During these UL motor tasks cortical motor region activation (fNIRS and EEG) and kinematics (hand, arm, and trunk) are simultaneously recorded using Lab Streaming layer technology (see “Availability of data and materials” section for more details)

In the seated arm reaching task (see Fig. 5a), patients will be comfortably seated on a chair with armrests. A target is located in front of them (centred in relation to the body) at a distance such that the base of the metacarpal of the index finger touches the target when the elbow is extended, the shoulder at ~ 30° of flexion and the back resting on the chair. Patients will have to reach the target in two conditions: (i) spontaneous condition, where patients are free to use trunk flexion compensation as they spontaneously do, and (ii) maximal condition, where patients limit trunk movements with the help of a light tactile feedback from the therapist to minimize trunk movements. A practice trial will be done to familiarize the patients on the timing of reaching (~ 5 reaches/20 s based on pilot testing) by following a recorded voice of investigator to start and return phase of the reaching movement. After a 2-min rest period, the patients will be required to repeat the reaching movements first with the paretic UL then with the non-paretic UL over 3 trials (20 s with 20 s rest between trials). The spontaneous condition will be undertaken first followed by the maximal condition. Kinect V2 (Microsoft, USA) and LSL-Kinect software (see “Availability of data and materials” for details) will be used to stream and analyse the UL kinematics to calculate PANU score and other kinematic variables according the methods outlined in our previous work [4].

#### Other/exploratory outcome measures

##### Functional motor tasks

The experimental setup for the Seated Reaching and Circular Steering tasks will allow synchronous measurements of motor cortical region activation (functional near infrared spectroscopy-fNIRS and electroencephalography-EEG) and UL kinematics using lab streaming layers (LSL, https://github.com/labstreaminglayer/App-LabRecorder).

In the circular steering task (see Fig. 5b), a computerized version of a circular steering task (LSL-Mouse; see “Availability of data and materials” for details) based on the speed–accuracy trade-off [8] will be used to determine performance improvements of the paretic UL. Patients will be comfortably seated in front of a table with a graphical tablet (A3 size, Wacom, Kazo, Japan) and stylus moulded onto a mouse pad for ease of hand placement for stroke patients. A 24-in. display screen placed behind the tablet will show the circular tunnel and a cursor matching the location of the stylus on the tablet. The patient’s hand will be placed at a location of the tablet corresponding to the top of the circle tunnel allowing comfortable elbow extension without trunk flexion from the chair. The instruction to the patient will be to move the cursor within the circular tunnel for 20 s, as fast as possible and some errors hitting the tunnel walls are allowed. Biasing speed over accuracy of movements is emphasized in the circular steering task to allow maximum potential of patients to gain benefit from andodal tDCS of the ipsilesional motor cortex [8, 31]. A practice trial will be provided to the patient where they will be able to see their errors online and movement path such that the investigator will inform the patient to speed up the movement if errors are less than 15% (based on pilot testing). After a 2-min rest period, the circular steering task will be repeated over 3 trials (20-s task interspaced by 20-s rest) first with the paretic UL followed by the non-paretic UL.

In the index of performance (IP), speed and accuracy will be calculated as in our previous work [8]. Additionally, trunk displacements will be recorded using the LSL-Kinect and trunk compensation will be determined.

In the Armeo Ladybug game (see Fig. 4), the Ladybug game on the Armeo exoskeleton will allow changes UL movement performance and motor cortical region activation to be determined from a task that will be used during the 3-week combined VRT and anodal HD-tDCS program. The ladybug task will require the patient to “catch” a series of 48 Ladybugs appearing one after the other at different positions on the display screen in less than 3 min. The UL kinematics will be recorded by the Armeo software, which will be analysed to determine the variability trajectory of the end effector to reach the target. Performance metric of the speed and smoothness of UL movements will be based on our previous work [64].

##### Motor Cortical region activation (fNIRS/EEG)

**Fig. 6:**
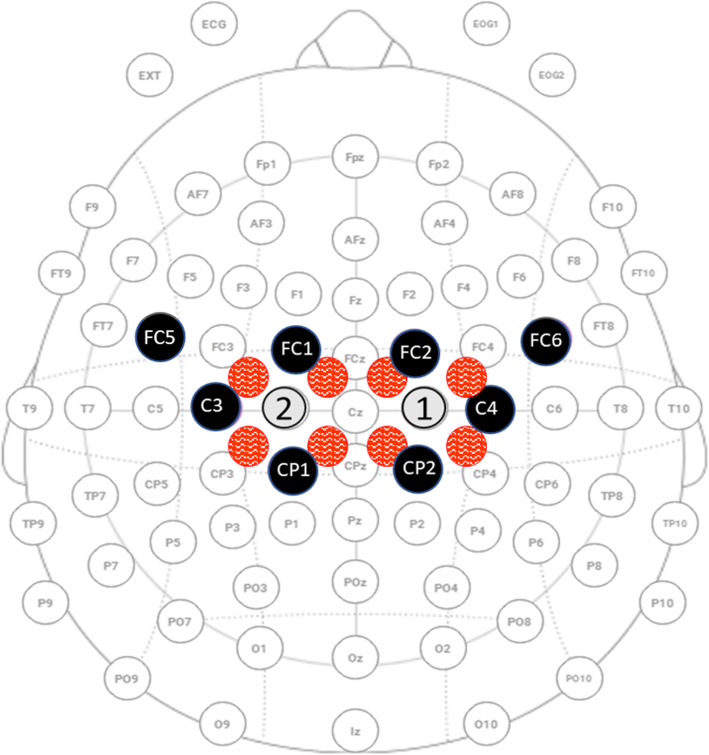
Locations of the electroencephalography (EEG) electrodes and functional near-infrared spectroscopy (fNIRS) probes over the bihemispheric motor cortical regions. The Starstim fNIRS integration (Starstim8-Octamon+) allowed for 8 EEG (4 electrodes [black circles] on the left [C3, FC5, FC1, CP1] and right [C4, FC2, FC6, and CP2] hemispheres) electrodes and 8 fNIRS (4 fNIRS transmitters (red circles) located ~ 3 cm from a receiver (numbered 1, 2) on each hemisphere) channels

Ipsilesional and contralesional motor cortical region activation will be measured during the 3 functional motor tasks (Seated reaching task, Circular steering task, and Armeo ladybug game) using a Starstim fNIRS integration (Starstim8, Neuroelectrics, Barcelona, Spain; Octamon+, Artinis Medical Systems, the Netherlands) that allows combined EEG and fNIRS measurements with the same neoprene cap used for the HD-tDCS setup. The 8 EEG electrodes and 8 fNIRS channels will be configured above the left and right motor cortical region (around C3 and C4 electrode locations, respectively on a 10–20 system; see Fig. 6).

EEG measures functional brain activity by detecting the variations of electrical fields created by neuronal activation, while fNIRS allows indirect measurement of neuronal activity by monitoring local changes in the oxygenated (O_2_Hb) and deoxygenated (HHb) haemoglobin concentrations in the cerebral microcirculation based on neurovascular coupling mechanism [2, 59]. These two imaging methods have previously been independently used to evaluate changes in motor cortical region activation during functional movements following stroke rehabilitation programs [7, 18], and fNIRS has already been tested and validated during various UL tasks by our team (reaching task in [6]; force task in [18] and in [20]).

In fNIRS analysis, changes in the average, peak, and area under the curve values of the concentration of O_2_Hb and HHb in the ipsilesional and contralesional motor cortical region will be determined during the 3 functional UL movement tasks. In addition, a laterality index (LI) will be calculated for each task and trial [20] to determine whether sensorimotor cortex activation, based on O_2_Hb concentration, during the task is mainly contralesional or ipsilesional. The LI will be calculated from the ratio of ipsilesional and contralesional O_2_Hb concentration, which varies between − 1 (purely contralesional activation) and + 1 (purely ipsilesional activation [18]).

In EEG analysis, changes in sensorimotor cortex neural oscillations will be measured by the magnitude and ratio of alpha- and beta-frequency power in both ipsilesional and contralesional motor cortical regional at rest and during the 3 functional UL movement tasks. The event-related desynchronization (ERD) of the alpha-motor mu rhythm (8–12 Hz) and of the low beta rhythm (12–20 Hz) will be calculated at the start of each trial. These specific band frequencies are well known to be involved during UL movement for healthy subjects but also for stroke patients [7].

##### Pain and fatigue of the paretic upper limb

Before and after each VRT session, pain and fatigue of the paretic UL during movement will be collected using a visual analogue scale (VAS). The VAS consists of a straight line measuring 10 cm, with 0 cm corresponding to the absence of pain/fatigue and 10 cm corresponding to maximum pain/fatigue.

##### Perceived exertion

Perceived difficulty will be evaluated after each VRT session using the Borg scale of perceived exertion [12]. The Borg scale extends from 6 “no feeling of exertion,” to “very, very hard,” which rates a 20.

### Data management and monitoring

During the trial, the sponsor of the project, Montpellier University Hospital (contact: Director of Innovation and Research Department) will be responsible of monitoring the study.

#### Collecting and data management

The participant will only be identified by a unique identification number, the first letter of the name, the first letter of the first name, his gender, and the year of birth. An identification list of subjects will be kept in the investigator’s file. The investigator will ensure that the anonymity of each person participating in the study is guaranteed. Information will be collected for each participant in a standardized observation booklet filled out by the investigator or the co-investigator.

The source documents are the original documents, data, and files, from which the data concerning the research participants are entered in the electronic case report form (eCRF) by dedicated employees at Montpellier University Hospital. The investigator undertakes to authorize direct access to the source data of the study during control, audit, or inspection visits. Data from the actimetry, from the Armeo and from the functional motor tasks (kinematics, performance, EEG/fNIRS) will be extracted from the local password-protected computers and stored on the EuroMov centre private server that can be accessed only by the registered investigators (KB, CM, DM, MM, SP, PJ, GD) with reinforced authentication during the duration of the project. All data will be checked monthly by the PI to ensure that all protocols and ethical guidelines for data collection and analysis are followed.

Study data are entered through the e-CRF developed using ENNOV CLINICAL software, which allows real-time data quality control. In order to meet regulatory requirements, this software complies with the recommendations concerning computerized systems for the management of clinical trials and electronic signature and standards. Connection is made by a unique password and identifier specific to each user, which will only give him access to his patients’ data. An audit function is integrated into the software, allowing traceability of the data collected as well as the modifications made. The encrypted data will then be transmitted to the department responsible for data management via a secure internet connection. The eCRF must consist of all the information required by the protocol.

The individual data necessary for the analysis of the study will be:

- Entered into e-CRFs as they are obtained, whether they are clinical or para-clinical data
- Anonymized by the investigator (the participant will be identified only by a unique identification number, the first letter of the name, the first letter of the first name, and the year of birth; an identification list of subjects will be kept in the investigator’s file)
- Authenticated by an electronic signature of the investigator
- All entered; therefore, the missing data must be justified

First, the data entered in the e-CRF is checked and validated by the Clinical Research Associate (CRA) from the source documents. Secondly, the data manager performs additional computerized consistency tests based on the presence of non-standard, missing, aberrant, or inconsistent data and which are performed regularly during patient recruitment and follow-up. All consistency tests are defined in advance in specifications proposed by the data manager and validated by the investigator, the statistician, and the CRA. Each inconsistency identified on the eCRF (“Queries”) is the subject of a request for correction or justification from the investigator (response to “Queries”). The latter undertakes to make himself available to the members of the research team and to provide them with all the additional information required to resolve these errors. This information is reported in the study database. Once the study is complete, namely the data required for the protocol and any additional data entered (self-questionnaires), the data monitored and validated, the study database is frozen.

The maintenance of the database is the responsibility of the Clinical and Epidemiology Research Unit of the Innovation and Research Department of the study sponsor. The data is kept in an ASCII type format.

The data encoding is integrated into eCRFs. The computer files and the follow-up of any modification are saved and made available by the Innovation and Research Department of Montpellier University Hospital. The database freezing is carried out according to the Innovation and Research Department procedures. The freeze is documented by a database freeze certificate. It takes place once all the data is verified and the corrections requested from the investigators are obtained.

The closure of the trial including the closure of the centres will be carried out in accordance with Good Clinical Practice and International Conference on Harmonization. The medical, administrative, and observation records (all study-related documents) will be kept for the duration of the study, then archived in locked filing cabinets at the PRM department of Montpellier University Hospital, for a minimum period of 15 years after the end of the project.

#### Monitoring and quality control

The project will be monitored by the CRA. Appropriate monitoring will be implemented based on a risk grid related to the project. In accordance with this grid, the CRA will carry out regular follow-ups of the project’s investigation centres (establishment visit, follow-up according to the rhythm of inclusions and a closing visit). All checks will be the subject of a monitoring report by written report (traceability of visits). The investigators agreed to comply with the requirements of the sponsor and the competent authority with regard to an audit or inspection of the research. The audit might be applied to all stages of research, from the development of the protocol to the publication of results and the classification of data used or produced in the context of the research.

### Adverse events management

Any adverse events (i.e., muscle or joint pain, syncope, falls and injuries) or serious adverse events (SAEs, i.e., any incident requiring hospitalization) during the HD-tDCS/VRT sessions or testing protocol will be reported to an investigator by filling out the Incident Report Form (IRF). All adverse events will be also recorded, treated, and evaluated upon signing of the consent and until the end of the patient’s participation in the study and/or the end of the collection of adverse events. The Innovation and Research Department will (1) assess the causal link between the serious adverse event and the research; (2) assess whether the adverse reaction is expected or unexpected, using the reference document, the protocol in force for this study; and (3) declare all serious and unexpected adverse reactions within the regulatory deadlines to the competent Health Authorities and the Ethics Committees concerned and informs the investigators at intervals appropriate to the research. Given the type of research (interventional research involving category 2 humans), no annual safety report is planned for this study and given the low risks added by the study, an independent oversight committee will not be established for this study.

### Statistical analysis

#### Sample size

Allman et al. [1] reported that compared to sham, real anodal tDCS (1 mA, conventional montage, ipsilesional motor cortex) combined with 9 days of UL rehabilitation in chronic stroke patients had a moderate effect size *d* = 0.4 on the WMFT at 3 months after the intervention. In the meta-analysis of Chhatbar et al. [16], UL motor recovery (FM-UE) in a chronic stroke population was superior in the group receiving a real tDCS (in comparison to sham) with higher current density and smaller electrode size (large effect size *g* = 1.23). Because we will use anodal HD-tDCS with a higher current density (2 mA) and longer rehabilitation dose (13 days) in chronic stroke patients, we estimate that our protocol should have an effect size of *d* = 0.8 on the WMFT at 3 months. Using an alpha bilateral risk of 5% and a power of 80%, 26 patients per group are needed for analysis. If we count a loss of 10% of the patients, we will have to recruit 58 patients (*n* = 29 per group).

#### Statistical method

Statistical analysis will be performed using the latest version of the free R software. A *p*-value of 0.05 will be considered significant for all analyses. All outcome measures will be assessed at V1, V2, and V3. The primary outcome will be the difference between both groups (*intervention, placebo*) at V3 on the WMFT. The secondary outcomes will be the changes between V1 and V2 (learning) and between V2 and V3 (retention) for both groups.

#### Descriptive analysis

Analysis will be done on the intent-to-treat sample population.

All patients’ characteristics at pre-test (V1), post-test (V2), and retention test (V3) will be described according to the randomization group. Qualitative variables will be described using frequency and percentages of each class. Quantitative variables will be described using median and quartiles.

#### Primary outcome measure analysis

The aim of the primary outcome analysis will be to compare the functional motor capacity of the UL (WMFT) in the intervention group comparatively to the placebo group at V3. The normality of the data from the WMFT will be analysed by the Shapiro-Wilk test. The variance homogeneity will be assessed using the Bartlett test. If the normality is respected, a parametric Student *t*-test will be performed for mean comparisons. If the distribution of the data is not normal, a non-parametric Wilcoxon signed-rank test will be used.

#### Secondary outcome measures analysis

The secondary outcome measures (FM-UE, BBT, PANU, BI, and actimetry) will be used to assess the changes in UL impairment, function, non-use, and ADL from V1 at inclusion to V2, and V2 to V3.

#### Other/exploratory outcome measures analysis

The outcome measures from the 3 functional motor tasks (Seated reaching, circular steering task, Armeo ladybug game) will be used to assess changes in UL performance (e.g., targets reached, IP, speed, accuracy, performance variability) and bihemispheric motor cortical regional activation (e.g., fNIRS-LI and EEG-ERD) from V1 to V2, and V2 to V3. Linear mixed models for the evolution of all these measures will be derived to assess the effect of time (V1, V2, V3) and of group (intervention, placebo). The interaction of group with time will be tested, along with the interaction of group with changes between V1 and V2 and between V2 and V3.

### Ethic and reglementary aspect

The research will be conducted in compliance with the French regulations in force, in particular the provisions relating to research involving humans: Law No. 2012-300 of 5 March 2012 relating to research involving humans and its implementing decrees (decree No. 2016-1537 of November 16, 2016, relating to research involving humans, decree No. 2017-884 of May 9, 2017, amending certain regulatory provisions relating to research involving humans), the laws of Bioethics (if applicable), Law No. 78-17 of January 6, 1978, as amended, relating to computing, files and freedoms, the Declaration of Helsinki, and good clinical practices. In the context of this research involving the category 2 human person, the authorization of the competent authority is not required. For information, the sponsor sends the favourable opinion of the CPP and the summary of the protocol to the competent authority.

Prior to carrying out this research involving the human person, the subject’s free, informed consent will be obtained after being informed, by the investigator during a consultation and after a sufficient period of reflection. The information intended for trial participants include all the elements defined in Law No. 2012-300 of 5 March 2012 relating to research involving the human person (Jardé Law) and is written in a simple manner, in language understandable by the participant.

The Montpellier University Hospital, sponsor of the study, has taken out a civil liability insurance contract for the entire duration of the study with Newline Syndicate 1218 at Lloyd’s guaranteeing its own civil liability as well as that of any party involved in the conduct of the trial, regardless of the nature of the links between the participants and the sponsor.

The data recorded during this research are subject to computer processing under the responsibility of the Montpellier University Hospital, the sponsor, in compliance with the law no. 78-17 of January 6, 1978, as amended relating to the IT, files, and freedoms.

## Discussion

The aim of the ReArm project is to evaluate the additional effects on paretic UL function by including anodal HD-tDCS during a 3-week intensive VRT program to supplement conventional therapy in late subacute and chronic stroke patients.

VRT and conventional therapy are equally effective in improving paretic UL function in stroke patients [45]; however, VRT is less labour intensive from a physiotherapy and occupational therapy perspective for the same clinical gains. So therapists can focus on other aspects of rehabilitation as such as providing encouragement, directions, and observing the quality of movement. As such, the PRM departments of Montpellier and Nimes University Hospitals have integrated VRT sessions into conventional therapy sessions as a standard in-patient stroke rehabilitation program. Anodal HD-tDCS is as a relatively simple and easily applied NIBS technique that has been shown to enhance motor cortical excitability and plasticity. Anodal HD-tDCS is an ideal candidate to help boost re-learning of paretic UL function in chronic stroke patients. Accordingly, combining anodal HD-tDCS with VRT rehabilitation could further improve neuroplastic effects induced by VRT alone and lead to greater and sustainable clinical gains [11].

The quadruple-blind, randomized, sham-controlled, bi-centre, two-arm parallel, interventional design of the ReArm project was implemented to ascertain whether adding anodal HD-tDCS, a more focalized and intensive form of anodal tDCS, during 3 weeks of VRT (13 × 30-min sessions) that complements conventional therapy (39 × 30-min sessions) would lead to greater and sustained improvements in paretic UL function. We are expecting a significantly improved functional recovery of the paretic UL for the group receiving real anodal HD-tDCS (*n* = 29) compared to the group receiving sham stimulation (*n* = 29). We will evaluate the effects of the ReArm rehabilitation program using various paretic UL assessments of (i) motor function (WMFT, FM-UE), (ii) prehension dexterity (BBT), (iii) non-use (PANU), (iv) everyday life activities (BI), and (v) motor performance (IP, speed, accuracy, compensation, variability). We will also determine pain, fatigue, and perceived exertion after each VRT session as well as neural biomarkers of paretic UL function by assessments of ipsilesional and contralesional motor cortical region plasticity (fNIRS-LI, amplitude, time to peak and EEG-alpha and beta ERD) during functional motor tasks. Although the primary outcome is based on the clinical assessment of the WMFT at 3 months after the intervention, the ReArm project’s multiple secondary and exploratory outcome measures allows to probe other aspects of paretic UL recovery that may benefit from the boost to neuroplasticity afforded by anodal HD-tDCS supplemented during the VRT sessions, which include novel assessments of paretic UL non-use (PANU), use-in ADL (actimetry), and performance (speed and accuracy), including a mechanistic understanding of such paretic UL improvements through neuroplasticity changes in ipsilesional motor cortical regional activation monitored using non-invasive and portable neuroimaging technologies (fNIRS and EEG).

With support from our primary and secondary outcome results, the impact of the ReArm project for the public health care system could be a better efficiency of paretic UL rehabilitation for chronic stroke patients at unchanged or lower cost. With combined HD-tDCS and VRT, we expect a better paretic UL recovery without an increased rehabilitation time commitment, without an increased human resource costs (e.g., physiotherapy and occupational therapy time) but with a relatively minor hardware related costs (tDCS device and consumables) costs. Moreover, this innovative treatment with HD-tDCS is adapted to a majority of patients, since tDCS allows to stimulate the patient while receiving rehabilitation treatment. Therefore, tDCS has a promising clinical potential in post-stroke rehabilitation [61, 63]. Thus, improving the efficiency of recovery for a majority of stroke patients may have an impact on patients’ health and on public health costs.

The benefit for the patients taking part of this study could be a better efficiency of the rehabilitation without changing the length of the rehabilitation programs. Indeed, the association of the two methods proposed (anodal HD-tDCS and VRT) could allow, for the same dose of rehabilitation, a better and faster recovery of the paretic UL function. Patients could thus enter in the virtual spiral of “use it and improve it” aided by the rehabilitation efficiency [38]. This could lead the patient to a better independence on daily life activities and to a better life quality.

### Dissemination plan

The results derived from the primary and secondary outcome analysis of this project will be reported in peer-reviewed journal articles and presented at leading national and international conferences. Results derived from the other/exploratory outcome analysis of only the baseline (V1) session will be included in the PhD thesis of CM. Results will be communicated to health professionals and participants through meetings and email/letter, respectively.

## Supplementary Information

**Additional file 1.** SPIRIT 2013 Checklist: Recommended items to address in a clinical trial protocol and related documents.

## Data Availability

The full unrestricted dataset will be given to the trial statistician of the project (CD) by the principal investigator (KB). Agreements with EuroMov-DHM laboratory will allow full access to SP for the EEG and fNIRS dataset and DM for the kinematics and actimetry dataset. CM and MM will have restricted access to the full dataset. GD, BX, and GF will have restricted access to EEG, fNIRS and kinematics dataset. The final datasets will be available from the corresponding author on reasonable request. The software applications developed for this project and the most recent version of the software are available from the corresponding author’s Github repository (LSL-Mouse, https://github.com/KarimaBak/LSL-Mouse, and LSL-KinectV2, https://github.com/KarimaBak/LSL-KinectV2) and an archived version on the Zenodo repository (LSL-Mouse, DOI 10.5281/zenodo.4297675; LSL-KinectV2, DOI 10.5281/zenodo.4300182). Other materials used in the project (e.g., consent forms) will be available from the corresponding author on reasonable request.

## Abbreviations

ADL: Activities of daily life
BI: Barthel Index
BBT: Box and Block test
EEG: Electroencephalography
FM-UE: Fugl-Meyer Upper Extremity
fNIRS: Functional near-infrared spectroscopy
HD-tDCS: High-definition transcranial direct current stimulation
NIBS: Non-invasive brain stimulation
PANU: Proximal Arm Non-Use
UL: Upper limb
VRT: Virtual reality therapy
WMFT: Wolf Motor Function Test

## References

[1] Allman C, Amadi U, Winkler AM, Wilkins L, Filippini N, Kischka U, Stagg CJ, Johansen-Berg H (2016). Ipsilesional anodal tDCS enhances the functional benefits of rehabilitation in patients after stroke. Sci Translat Med.

[2] Attwell D, Iadecola C. The neural basis of functional brain imaging signals. Trends Neurosci. 2002;25(12):621–5. 10.1016/s0166-2236(02)02264-6 12446129

[3] Bailey RR, Klaesner JW, Lang CE (2015). Quantifying real-world upper-limb activity in nondisabled adults and adults with chronic stroke: Neurorehabilitation and Neural Repair.

[4] Bakhti KKA, Laffont I, Muthalib M, Froger J, Mottet D (2018). Kinect-based assessment of proximal arm non-use after a stroke. J Neuroeng Rehab.

[5] Bakhti KKA, Mottet D, Schweighofer N, Froger J, Laffont I (2017). Proximal arm non-use when reaching after a stroke. Neurosci Lett.

[6] Bakhti K, Muthalib M, Perrey S, Froger J, Laffont I, Mottet D (2016). FNIRS provides clues about the neural correlates of the learned non-use of the paretic arm after a stroke. Ann Phys Rehab Med.

[7] Bartur G, Pratt H, Soroker N (2019). Changes in mu and beta amplitude of the EEG during upper limb movement correlate with motor impairment and structural damage in subacute stroke. Clin Neurophysiol.

[8] Besson P, Muthalib M, De Vassoigne C, Rothwell J, Perrey S (2020). Effects of multiple sessions of cathodal priming and anodal HD-tDCS on visuo motor task plateau learning and retention. Brain Sci.

[9] Besson P, Muthalib M, Dray G, Rothwell J, Perrey S (2019). Concurrent anodal transcranial direct-current stimulation and motor task to influence sensorimotor cortex activation. Brain Res.

[10] Bikson M, Grossman P, Thomas C, Zannou AL, Jiang J, Adnan T, Mourdoukoutas AP, Kronberg G, Truong D, Boggio P, Brunoni AR, Charvet L, Fregni F, Fritsch B, Gillick B, Hamilton RH, Hampstead BM, Jankord R, Kirton A, Knotkova H, Liebetanz D, Liu A, Loo C, Nitsche MA, Reis J, Richardson JD, Rotenberg A, Turkeltaub PE, Woods AJ (2016). Safety of transcranial direct current stimulation: evidence based update 2016. Brain Stimul.

[11] Bolognini N, Pascual-Leone A, Fregni F (2009). Using non-invasive brain stimulation to augment motor training-induced plasticity. J NeuroEng Rehab.

[12] Borg G (1970). Perceived exertion as an indicator of somatic stress. Scand J Rehab Med.

[13] Cabibel V, Muthalib M, Teo W-P, Perrey S (2018). High-definition transcranial direct-current stimulation of the right M1 further facilitates left M1 excitability during crossed facilitation. J Neurophysiol.

[14] Chen H-M, Chen CC, Hsueh I-P, Huang S-L, Hsieh C-L (2009). Test-retest reproducibility and smallest real difference of 5 hand function tests in patients with stroke. Neurorehab Neural Repair.

[15] Chhatbar PY, Chen R, Deardorff R, Dellenbach B, Kautz SA, George MS, Feng W (2017). Safety and tolerability of transcranial direct current stimulation to stroke patients – a phase I current escalation study. Brain Stimul.

[16] Chhatbar PY, Feng W. Data Synthesis in Meta-Analysis may Conclude Differently on Cognitive Effect From Transcranial Direct Current Stimulation. Brain Stimul. 2015;8(5):974–6. 10.1016/j.brs.2015.06.001 PMC13524162 26115775

[17] Chhatbar PY, Ramakrishnan V, Kautz S, George MS, Adams RJ, Feng W (2016). Transcranial direct current stimulation post-stroke upper extremity motor recovery studies exhibit a dose-response relationship. Brain Stimul.

[18] Delorme M, Vergotte G, Perrey S, Froger J, Laffont I (2019). Time course of sensorimotor cortex reorganization during upper extremity task accompanying motor recovery early after stroke: an fNIRS study. Restor Neurol Neurosci.

[19] Demain S, Burridge J, Ellis-Hill C, Hughes A-M, Yardley L, Tedesco-Triccas L, et al. Assistive technologies after stroke: self-management or fending for yourself? A focus group study. BMC Health Serv Res. 2013;13(1). 10.1186/1472-6963-13-334. 10.1186/1472-6963-13-334 PMC3765821 23968362

[20] Derosière G, Alexandre F, Bourdillon N, Mandrick K, Ward TE, Perrey S (2014). Similar scaling of contralateral and ipsilateral cortical responses during graded unimanual force generation. NeuroImage.

[21] Elsner B, Kugler J, Mehrholz J (2018). Transcranial direct current stimulation (tDCS) for upper limb rehabilitation after stroke: future directions. J Neuroeng Rehab.

[22] Elsner B, Kugler J, Pohl M, Mehrholz J (2020). Transcranial direct current stimulation (tDCS) for improving activities of daily living, and physical and cognitive functioning, in people after stroke. Cochrane Database Syst Rev.

[23] Figlewski K, Blicher JU, Mortensen J, Severinsen KE, Nielsen JF, Andersen H (2017). Transcranial direct current stimulation potentiates improvements in functional ability in patients with chronic stroke receiving constraint-induced movement therapy. Stroke.

[24] Fleming MK, Rothwell JC, Sztriha L, Teo JT, Newham DJ (2017). The effect of transcranial direct current stimulation on motor sequence learning and upper limb function after stroke. Clin Neurophysiol.

[25] Flöel A (2014). TDCS-enhanced motor and cognitive function in neurological diseases. NeuroImage.

[26] Fuentes MA, Borrego A, Latorre J, Colomer C, Alcañiz M, Sánchez-Ledesma MJ, Noé E, Llorens R (2018). Combined transcranial direct current stimulation and virtual reality-based paradigm for upper limb rehabilitation in individuals with restricted movements. A feasibility study with a chronic stroke survivor with severe hemiparesis. J Med Syst.

[27] Fugl-Meyer AR, Jääskö L, Leyman I, Olsson S, Steglind S (1975). The post-stroke hemiplegic patient. 1. A method for evaluation of physical performance. Scand J Rehabil Med.

[28] Gandiga PC, Hummel FC, Cohen LG. Transcranial DC stimulation (tDCS) : A tool for double-blind sham-controlled clinical studies in brain stimulation. Clin Neurophysiol. 2006;117(4):845–50. 10.1016/j.clinph.2005.12.003 16427357

[29] Gauthier L, Dehaut F, Joanette Y (1989). The Bells Test: a quantitative and qualitative test for visual neglect.

[30] Gladstone DJ, Danells CJ, Black SE (2002). The fugl-meyer assessment of motor recovery after stroke: a critical review of its measurement properties. Neurorehabil Neural Repair.

[31] Hamoudi M, Schambra HM, Fritsch B, Schoechlin-Marx A, Weiller C, Cohen LG, Reis J (2018). Transcranial direct current stimulation enhances motor skill learning but not generalization in chronic stroke. Neurorehabil Neural Repair.

[32] Hummel F, Celnik P, Giraux P, Floel A, Wu W-H, Gerloff C, Cohen LG (2005). Effects of non-invasive cortical stimulation on skilled motor function in chronic stroke. Brain.

[33] Jang SH, You SH, Hallett M, Cho YW, Park C-M, Cho S-H, Lee H-Y, Kim T-H (2005). Cortical reorganization and associated functional motor recovery after virtual reality in patients with chronic stroke: an experimenter-blind preliminary study. Arch Phys Med Rehabil.

[34] Jones TA (2017). Motor compensation and its effects on neural reorganization after stroke. Nature Reviews. Neuroscience.

[35] Kalafat M, Hugonot-Diener L, Poitrenaud J (2003). Standardisation et étalonnage français du « Mini Mental State » (MMS) version GRECO. Rev Neuropsycol.

[36] Kaplan E, Goodglass H, Weintraub S, Goodglass H (1983). Boston naming test. Lea & Febiger.

[37] Kleim JA (2011). Neural plasticity and neurorehabilitation: teaching the new brain old tricks. J Commun Disord.

[38] Kleim JA, Jones TA (2008). Principles of experience-dependent neural plasticity: implications for rehabilitation after brain damage. J Speech Lang Hear Res.

[39] Kuo H-I, Bikson M, Datta A, Minhas P, Paulus W, Kuo M-F, Nitsche MA (2013). Comparing cortical plasticity induced by conventional and high-definition 4 × 1 ring tDCS: a neurophysiological study. Brain Stimul.

[40] Kwakkel G, Kollen B, Lindeman E (2004). Understanding the pattern of functional recovery after stroke: facts and theories. Restor Neurol Neurosci.

[41] Laffont I, Bakhti K, Coroian F, van Dokkum L, Mottet D, Schweighofer N, Froger J (2014). Innovative technologies applied to sensorimotor rehabilitation after stroke. Ann Phys Rehabil Med.

[42] Laffont I, Froger J, Jourdan C, Bakhti K, van Dokkum LEH, Gouaich A, Bonnin HY, Armingaud P, Jaussent A, Picot MC, Le Bars E, Dupeyron A, Arquizan C, Gelis A, Mottet D (2020). Rehabilitation of the upper arm early after stroke: video games versus conventional rehabilitation. A randomized controlled trial. Ann Phys Rehabil Med.

[43] Lang CE, Macdonald JR, Reisman DS, Boyd L, Jacobson Kimberley T, Schindler-Ivens SM, Hornby TG, Ross SA, Scheets PL (2009). Observation of amounts of movement practice provided during stroke rehabilitation. Arch Phys Med Rehabil.

[44] Lang CE, Waddell KJ, Klaesner JW, Bland MD (2017). A method for quantifying upper limb performance in daily life using accelerometers. J Visual Exper.

[45] Laver KE, Lange B, George S, Deutsch JE, Saposnik G, Crotty M (2017). Virtual reality for stroke rehabilitation. Cochrane Database Syst Rev.

[46] Leuenberger K, Gonzenbach R, Wachter S, Luft A, Gassert R (2017). A method to qualitatively assess arm use in stroke survivors in the home environment. Med Biol Eng Comput.

[47] Lin J-H, Hsu M-J, Sheu C-F, Wu T-S, Lin R-T, Chen C-H, Hsieh C-L (2009). Psychometric comparisons of 4 measures for assessing upper-extremity function in people with stroke. Phys Ther.

[48] Lindberg P, Uppsala universitet, & Medicinska fakulteten (2007). Brain plasticity and upper limb function after stroke: some implications for rehabilitation. Acta Universitatis Upsaliensis: Univ.-bibl. [distributör]..

[49] Lo HS, Xie SQ (2012). Exoskeleton robots for upper-limb rehabilitation: state of the art and future prospects. Med Eng Phys.

[50] Lum PS, Mulroy S, Amdur RL, Requejo P, Prilutsky BI, Dromerick AW. Gains in upper extremity function after stroke via recovery or compensation: potential differential effects on amount of real-world limb use. Top Stroke Rehabil. 2009;16(4):237–53. 10.1310/tsr1604-237. 10.1310/tsr1604-237 19740730

[51] Mahoney FI, Barthel DW (1965). Functional evaluation: the Barthel Index. Maryland State Med J.

[52] Mathiowetz V, Volland G, Kashman N, Weber K. Adult norms for the box and block test of manual dexterity. Am J Occup Ther. 1985;39:386–91. 10.5014/ajot.39.6.386 3160243

[53] Merlo A, Longhi M, Giannotti E, Prati P, Giacobbi M, Ruscelli E, Mancini A, Ottaviani M, Montanari L, Mazzoli D (2013). Upper limb evaluation with robotic exoskeleton. Normative values for indices of accuracy, speed and smoothness. NeuroRehabil.

[54] Miller EL, Murray L, Richards L, Zorowitz RD, Bakas T, Clark P, Billinger SA, on behalf of the American Heart Association Council on Cardiovascular Nursing and the Stroke Council (2010). Comprehensive overview of nursing and interdisciplinary rehabilitation care of the stroke patient: a scientific statement from the American Heart Association. Stroke.

[55] Muthalib M, Besson P, Rothwell J, Perrey S (2018). Focal hemodynamic responses in the stimulated hemisphere during high-definition transcranial direct current stimulation: focal hemodynamic responses during HD-tDCS. Neuromodulation.

[56] Nitsche MA, Paulus W (2011). Transcranial direct current stimulation—update 2011. Restor Neurol Neurosci.

[57] Page SJ, Fulk GD, Boyne P (2012). Clinically important differences for the upper-extremity Fugl-Meyer scale in people with minimal to moderate impairment due to chronic stroke. Phys Ther.

[58] Pan Y, Goodwin B, Sabelhaus E (2020). Feasibility of using acceleration-derived jerk to quantify bimanual arm use. J NeuroEngineering Rehabil.

[59] Perrey S. Non-invasive NIR spectroscopy of human brain function during exercise. Methods (San Diego, Calif.). 2008;45(4):289–99. 10.1016/j.ymeth.2008.04.005 18539160

[60] Pollock, A., Farmer, S. E., Brady, M. C., Langhorne, P., Mead, G. E., Mehrholz, J., & van Wijck, F. (2014). Interventions for improving upper limb function after stroke. In The Cochrane Collaboration (Éd.), Cochrane Database of Systematic Reviews. John Wiley & Sons, Ltd., DOI: 10.1002/14651858.CD010820.pub2 10.1002/14651858.CD010820.pub2 PMC6469541 25387001

[61] Reckow J, Rahman-Filipiak A, Garcia S, Schlaefflin S, Calhoun O, DaSilva AF, Bikson M, Hampstead BM (2018). Tolerability and blinding of 4x1 high-definition transcranial direct current stimulation (HD-tDCS) at two and three milliamps. Brain Stimul.

[62] Schabrun SM, Chipchase LS (2012). Priming the brain to learn: the future of therapy?. Manual Ther.

[63] Schlaug G, Renga V, Nair D (2008). Transcranial direct current stimulation in stroke recovery. Arch Neurol.

[64] Schweighofer N, Wang C, Mottet D, Laffont I, Bakhti K, Reinkensmeyer DJ, Rémy-Néris O (2018). Dissociating motor learning from recovery in exoskeleton training post-stroke. J NeuroEng Rehabil.

[65] Subramanian SK, Lourenço CB, Chilingaryan G, Sveistrup H, Levin MF (2013). Arm motor recovery using a virtual reality intervention in chronic stroke: randomized control trial. Neurorehabil Neural Repair.

[66] Subramanian SK, Prasanna SS (2018). Virtual reality and noninvasive brain stimulation in stroke: how effective is their combination for upper limb motor improvement?-A meta-analysis. PM R.

[67] Sveistrup H (2004). Motor rehabilitation using virtual reality. J NeuroEng Rehabil.

[68] Taub E, Uswatte G, Mark VW, Morris DMM (2006). The learned nonuse phenomenon: implications for rehabilitation. Europa Medicophysica.

[69] Taveggia G, Borboni A, Salvi L, Mulé C, Fogliaresi S, Villafañe JH, Casale R (2016). Efficacy of robot-assisted rehabilitation for the functional recovery of the upper limb in post-stroke patients: a randomized controlled study. Eur J Phys Rehabil Med.

[70] Teo W-P, Muthalib M, Yamin S, Hendy AM, Bramstedt K, Kotsopoulos E, Perrey S, Ayaz H (2016). Does a combination of virtual reality, neuromodulation and neuroimaging provide a comprehensive platform for neurorehabilitation? - A narrative review of the literature. Front Human Neurosci.

[71] Triccas LT, Burridge JH, Hughes A, Verheyden G, Desikan M, Rothwell J (2015). A double-blinded randomised controlled trial exploring the effect of anodal transcranial direct current stimulation and uni-lateral robot therapy for the impaired upper limb in sub-acute and chronic stroke. NeuroRehabil.

[72] Turolla A, Dam M, Ventura L, Tonin P, Agostini M, Zucconi C, Kiper P, Cagnin A, Piron L (2013). Virtual reality for the rehabilitation of the upper limb motor function after stroke: a prospective controlled trial. J NeuroEng Rehabil.

[73] Veerbeek JM, van Wegen E, van Peppen R, van der Wees PJ, Hendriks E, Rietberg M, Kwakkel G (2014). What is the evidence for physical therapy poststroke? A systematic review and meta-analysis. PLoS ONE.

[74] Wolf SL, Catlin PA, Ellis M, Archer AL, Morgan B, Piacentino A (2001). Assessing Wolf motor function test as outcome measure for research in patients after stroke. Stroke.

